# Evaluation of blood pressure and NT-proBNP in pugs with and without clinical signs of Brachycephalic Obstructive Airway Syndrome

**DOI:** 10.3389/fvets.2022.1015157

**Published:** 2022-12-22

**Authors:** Rebekka Mach, Pia Saskia Wiegel, Jan-Peter Bach, Martin Beyerbach, Charanthorn Levicar, Ingo Nolte

**Affiliations:** ^1^Clinic for Small Animals, University of Veterinary Medicine Hannover, Foundation, Hannover, Germany; ^2^Institute for Biometry, Epidemiology and Information Processing, University of Veterinary Medicine Hannover, Foundation, Hannover, Germany

**Keywords:** NT-proBNP, blood pressure, pug, Brachycephalic Obstructive Airway Syndrome (BOAS), cardiac biomarker

## Abstract

Brachycephalic Obstructive Airway Syndrome (BOAS) is a pathologic condition of the upper airways, frequently occurring in dogs of brachycephalic breeds including pugs. It has been suspected that BOAS may be associated with cardiovascular changes and an increased risk for hypertension. The cardiac biomarker NT-proBNP can help to differentiate cardiac from non-cardiac respiratory distress. A possible influence of BOAS on NT-proBNP values has not been investigated, however. The aim of the current study was to examine blood pressure and NT-proBNP levels in pugs with and without clinical signs of BOAS and compare them to values of mesocephalic dogs. For this purpose, NT-proBNP values of 42 pugs and six mesocephalic dogs and blood pressure measurements of 34 pugs and four mesocephalic dogs were explored in the present study. Pugs were examined for clinical signs of BOAS at rest and after a submaximal fitness test, and a functional BOAS grading was applied. Blood pressure (BP) was measured at the beginning and end of the study day and NT-proBNP values were obtained before and after exercise. Measured values of pugs with different degrees of clinical impairment due to BOAS were compared among each other as well as to the CG. In terms of systolic, mean, diastolic BP, and NT-pro BNP, there were no relevant differences between pugs and the CG and no obvious connection between the severity of BOAS symptoms and measured values. BP values of all groups were lower at the second measurement at the end of the study day. NT-proBNP measurements were higher after exercise. BP and NT-proBNP values in all groups were in agreement with commonly used reference ranges. In conclusion, the study adds evidence, that BP and NT-proBNP values did not differ between mesocephalic dogs and pugs with different levels of severity of BOAS but between the measurement times. Thus, in the present study, excitement and exercise seemed to have a greater influence on BP and NT-proBNP values than presence of BOAS symptoms or breed. Discovered values show that the commonly used reference ranges for BP and NT-proBNP are applicable in pugs. This indicates that NT-proBNP can be used to differentiate between cardiac and non-cardiac respiratory distress even in pugs with clinical symptoms of BOAS.

## 1. Introduction

Due to their specific anatomy, brachycephalic breeds, such as the pug, have an increased risk of developing various diseases and often suffer from the so-called Brachycephalic Obstructive Airway Syndrome (BOAS) (1–5). There is a paucity of literature regarding the impact of brachycephaly and BOAS on the cardiovascular system. Studies have investigated differences between brachycephalic and mesocephalic dogs in echocardiography (6, 7), blood pressure (8, 9), and cardiac biomarkers (10). However, studies concerned with measuring blood pressure (BP) and the cardiac biomarker N-terminal pro-B-Type natriuretic peptide (NT-proBNP) in pugs with different severity of BOAS symptoms are lacking.

Regarding BP, there are two studies in which dogs of different brachycephalic breeds had significantly higher BP values than mesocephalic dogs (8, 9). In two other studies, no significant differences between brachy- and mesocephalic dogs could be found (6, 11). A mechanism similarity to obstructive sleep apnoea (OSA) in humans is presumed as a possible reason for higher BP in brachycephalic dogs (8, 9). OSA in humans is a sleep-related breathing disorder associated with disruptions in sleep, hypoxemia, hypercapnia, and an increased risk for hypertension (12–14). A sleep-related breathing disorder showing similarities to OSA in humans was found in English Bulldogs, which therefore were described as a natural model for OSA (15). Brachycephaly is also listed as a risk factor for the development of hypertension in dogs in the current American College of Veterinary Internal Medicine (ACVIM) consensus statement on the identification, evaluation, and management of systemic hypertension in dogs and cats (16).

Cardiac biomarkers are a subject of growing interest in veterinary medicine and can assist in the diagnosis and prognosis of heart diseases (17–21). The cardiac biomarker NT-proBNP is one of the most important cardiac natriuretic peptides in dogs and a marker of cardiac wall stress (22, 23). Various studies showed that NT-proBNP could be used to differentiate between cardiac and non-cardiac respiratory distress (17, 22, 24–28). In one of these studies, BOAS is mentioned as a non-cardiac cause of respiratory distress (22), although the connection between BOAS and NT-proBNP levels has not been the subject of further investigation.

Breed-related influence on cardiac biomarkers, such as NT-proBNP, has already been demonstrated (29), but a breed-specific examination for pugs is still lacking. Investigations into breed-specific NT-proBNP levels in pugs might be helpful due to the prevalence of BOAS-related respiratory disorders in this breed, which might influence NT-proBNP levels.

NT-proBNP has already been shown to be increased after exercise (21) and was therefore measured at rest and after a submaximal fitness test (FT) to point out possible differences. Since clinical signs of BOAS increase under exercise conditions as well (30–32), investigations into the impact of exercise on NT-proBNP may clarify its efficiency in detecting relations to BOAS. In brachycephalic dogs with clinical symptoms of BOAS, the cardiac biomarker cardiac troponin (cTnI) was investigated and found to be increased in 47.8% of the study population (10). However, this was not the case for any of the 11 pugs, which had been included in the study (10).

The objective of the present study was to explore BP and NT-proBNP in pugs from a regular population of patients and to compare values with mesocephalic dogs. To differentiate between BOAS-related increases in BP and NT-proBNP levels, pugs were allocated to groups with different levels of severity of BOAS depending on their clinical presentation.

## 2. Materials and methods

The study protocol was approved by an institutional Ethics Commission (Lower Saxony State Office for Consumer Protection and Food Safety (LAVES), Oldenburg, Germany, 33.19-42502-05-19A424) and every dog owner had to sign a consent form.

### 2.1. Study animals and examinations

This prospective study was performed at the Clinic for Small Animals at the University of Veterinary Medicine, Foundation, Hannover, Germany between July 2019 and August 2020. All dogs participating in the study were privately owned. Pugs were eligible for study inclusion if they were at least 2 years old, had no previous upper airway surgery or relevant systemic diseases apart from possible BOAS. Mesocephalic dogs of different breeds served as control group (CG), with the same inclusion criteria applying to them. For a good comparability between the study groups, dogs of a similar age and weight range were chosen as CG.

Physical examination, echocardiography, blood count, and serum biochemical analysis were performed in each dog. Cardiac ultrasound was performed by an experienced investigator (JPB) using ultrasonic devices (Vivid E7 or E9, General Electrics, Inc., Boston, MA, USA). Dogs were excluded from the evaluation of BP or NT-proBNP if they showed a pathologic heart murmur in the physical examination, non-sinus arrhythmia or significant cardiac abnormalities identified on two dimensional (2D), M-Mode, and/or Doppler echocardiography. LA/Ao (ratio of diameters of left atrium and aortic root) and LVIDd (diastolic diameter of left ventricle) were assessed as described previously (33, 34). Blood count and serum biochemical analysis were performed to rule out systemic diseases, which could affect the results of BP or NT-proBNP measurements.

In total, 62 pugs and ten mesocephalic dogs serving as CG were included in the study. Of these, 11 pugs and three dogs in the CG were excluded from the evaluation of BP and NT-proBNP because they did not meet the inclusion criteria in the echocardiographic examination. Due to unwillingness to run on the treadmill, another eight pugs had to be excluded. Since dogs were required to have values for BP and NT-proBNP at both examination points for comparison, another nine pugs and three dogs in the CG were excluded from final calculations of BP because adequate values were only available in one of two measurement times. For NT-proBNP, this was the case in one pug and one dog in the CG. A flow chart giving an overview of the exclusion can be found in the (Supplementary Figure S1).

### 2.2. Blood pressure

BP was indirectly measured using an automated High Definition Oscillometry device (HDO, S+B med Vet GmbH, Babenhausen, Germany). Measurements were directly visualised on a tablet with the MDSWIN Software Analyses (S+B med Vet GmbH) and thus examined for possible errors like movement artifacts. Regarding the recommendations of the ACVIM, the first measurement was discarded and once reliable, consistent readings had been obtained, five to seven consecutive, consistent measurements were taken (16). A detailed protocol, based on the recommendations of the ACVIM, was completed with additional information like size of the cuff, owner presence, position, and stress level of each dog. Systolic, mean, and diastolic arterial blood pressure values (SBP, MBP, DBP, respectively) as well as pulse frequency were obtained. BP measurements were taken at two different times. The first BP measurement was performed during echocardiographic examination, which was performed as one of the earliest examinations. The second BP measurement was performed at the end of the study day in a quiet environment after the dogs had had a sufficient amount of time to recover from the previous examinations. Only dogs (*n* = 38) for which reliable measurement results had been obtained at both examination times were included in the final evaluation.

### 2.3. NT-proBNP

Blood was collected in EDTA-tubes from the vena saphena or vena cephalica antebrachii. Samples were taken before and after an FT. Plasma was obtained by centrifugation and the samples were stored at −80°C until shipment on dry ice to the laboratory (IDEXX Laboratories, Ludwigsburg, Germany). NT-proBNP was measured from 0.3 ml EDTA-Plasma by canine Cardiopet^®^ proBNP ELISA (IDEXX Laboratories).

### 2.4. Submaximal fitness test

The FT was performed on a motorised treadmill (“quasar,” h/p/cosmos sports and medical GmbH, Nussdorf-Traunstein, Germany). During the FT, dogs trotted 15 min at their individual comfort speed (four to eight kilometres per hour) on the treadmill with a measurement break of 1 min after 5–10 min. With the measurement breaks, the FT lasted 17 min in total.

### 2.5. Functional BOAS grading

A functional grading system, originally designed and validated by Liu et al. (35), was modified and applied to the dogs based on the findings after 15 min of exercise (35). It has been previously used in various studies investigating BOAS (30, 31, 36, 37). The dogs were classified as having no (grade 0), mild (grade 1), moderate (grade 2) or severe (grade 3) signs of BOAS by evaluating respiratory noises and inspiratory effort before and after exercise and possible signs of dyspnoea or cyanosis (Supplementary Table S1).

### 2.6. Statistical analysis

The study was analysed within an exploratory approach and only descriptive statistics were presented. For statistical analyses, SAS 9.4 and SAS Enterprise Guide 7.1 (SAS Institute, Inc., Cary., NC, USA) were used. Graphics were created with GraphPad Prism (GraphPad, San Diego, CA, USA).

## 3. Results

### 3.1. Study animals

A total of 62 pugs and 10 mesocephalic dogs serving as CG were examined. For final evaluation of BP, measurements of 34 pugs and four dogs in the CG could be included. For evaluation of NT-proBNP, values of 42 pugs and six dogs in the CG were taken for analysis. A detailed overview of baseline characteristics of included dogs can be found in Table 1.

**Table 1:** Baseline characteristics and running speed of the control group (CG) and pugs subdivided into BOAS grades 0–3 included in the evaluation of NT-proBNP and blood pressure.

**Parameter**	**Baseline characteristics**	**Groups**				
		**CG**	**Pugs**			
			**Grade 0**	**Grade 1**	**Grade 2**	**Grade 3**
NT-proBNP	Number (*n*)	6	9	6	21	6
	Gender (male/female)	4/2	6/3	2/4	10/11	2/4
	Age (years, mean ± SD)	6.0 ± 2.7	4.4 ± 1.6	3.6 ± 1.4	4.4 ± 2.5	4.6 ± 2.1
	Weight (kg, mean ± SD)	8.7 ± 1.9	9.6 ± 1.1	8.3 ± 0.9	9.1 ± 1.3	7.8 ± 1.5
	Speed (km/h, mean ± SD)	5.5 ± 0.8	5.1 ± 0.6	4.8 ± 0.4	5.0 ± 0.4	4.9 ± 0.6
Blood pressure	Number (*n*)	4	8	5	18	3
	Gender (male/female)	3/1	5/3	2/3	8/10	0/3
	Age (years, mean ± SD)	6.3 ± 3.1	4.4 ± 1.7	3.4 ± 1.3	4.5 ± 2.5	4.0 ± 2.2
	Weight (kg, mean ± SD)	9.9 ± 0.7	9.6 ± 1.2	8.4 ± 1.0	9.2 ± 1.4	7.3 ± 1.2
	Speed (km/h, mean ± SD)	5.7 ± 0.9	5.1 ± 0.6	5.0 ± 0.2	5.0 ± 0.4	4.6 ± 0.7

CG, control group; SD, standard deviation.

In the blood count and serum biochemical analysis, only few dogs showed minimal deviations from the reference values, which were therefore assumed to have no influence on BP and NT-proBNP (Supplementary Table S2).

### 3.2. Blood pressure

An overview of BP values of the CG and BOAS grades 0–3 pugs can be found in Table 2. Eleven dogs had SBP values between 140 and 160 mmHg (one in the CG, four in the BOAS grade 0 group, three in the BOAS grade 1 group, and three in the BOAS grade 2 group). None of the dogs had values above 160 mmHg. In all but three BP measurements, at least five readings were available. Due to the reason that for these three measurements at least three consecutive, consistent readings were available, they were also taken for evaluation.

**Table 2:** Mean (± SD) systolic (SBP), mean (MBP), diastolic blood pressure (DBP), and pulse frequency of dogs in the control group (CG) and pugs subdivided into BOAS grades 0–3 at first measurement time point during echocardiography^a^ and the second one at the end of the study day^b^.

**Variable/Groups**		**Pugs**			
	**CG (*n* = 4)**	**Grade 0**	**Grade 1**	**Grade 2**	**Grade 3**
		**(*n* = 8)**	**(*n* = 5)**	**(*n* = 18)**	**(*n* = 3)**
SBP^a^ (mmHg)	132.5 ± 18.9	134.5 ± 10.6	120.8 ± 11.7	132.2 ± 10.9	122.3 ± 3.8
MBP^a^ (mmHg)	90.8 ± 11.1	90.0 ± 8.8	86.4 ± 4.7	92.8 ± 9.0	88.3 ± 4.7
DBP^a^ (mmHg)	67.5 ± 8.4	65.8 ± 10.6	66.6 ± 3.0	71.2 ± 9.3	69.7 ± 4.9
Pulse frequency^a^	99 ± 22	97 ± 20	100 ± 27	102 ± 20	82 ± 4
SBP^b^ (mmHg)	119.0 ± 7.5	119.3 ± 8.4	115.0 ± 8.3	126.6 ± 13.8	113.7 ± 10.1
MBP^b^ (mmHg)	82.5 ± 4.2	82.6 ± 7.4	82.4 ± 7.4	88.2 ± 8.7	80.0 ± 7.0
DBP^b^ (mmHg)	62.5 ± 2.9	62.0 ± 8.2	64.4 ± 7.0	66.9 ± 7.1	61.0 ± 8.2
Pulse frequency^b^	91 ± 4	84 ± 27	101 ± 13	103 ± 17	100 ± 20

Since the first measurement was performed during echocardiography, all dogs (100%) lay in lateral recumbency. Thirteen percent of the dogs were relaxed, most patients (66%) slightly nervous, 18% were nervous, and 3% very nervous. During the second measurement, 16% lay in lateral recumbency, 37% in sternal recumbency, 31% were sitting and 16% standing. At that time, most dogs (84%) were relaxed and some (16%) were slightly nervous. No correlation between BP values with age, body weight, owner presence, position or stress level of the dogs was found.

### 3.3. NT-proBNP

**Figure 1:**
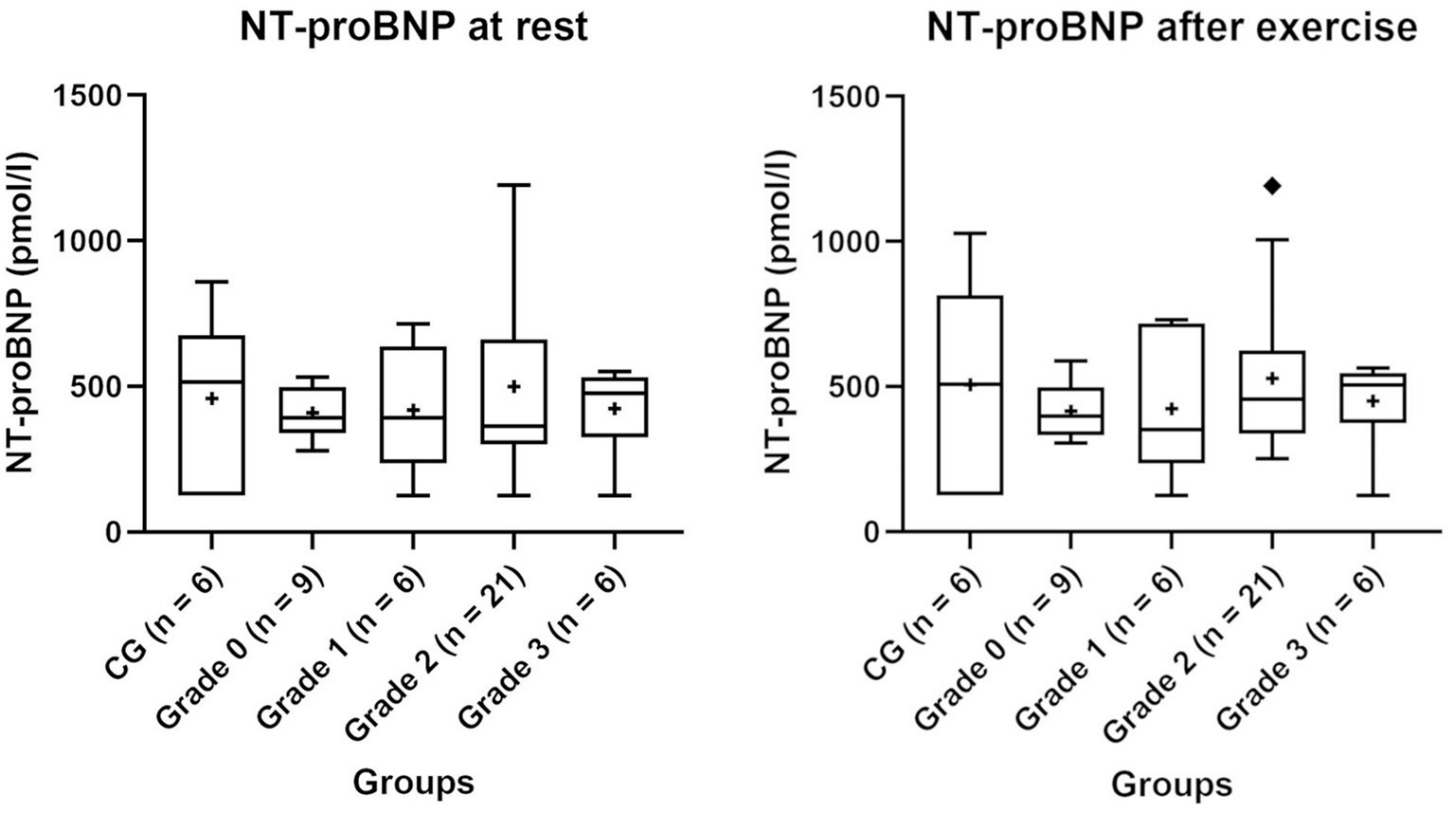
NT-proBNP values of the control group (CG) and groups of pugs subdivided into BOAS grades 0–3 at rest and after exercise. Boxes contain values from 1st to 3rd quartile, lines inside boxes indicate median values, + inside the boxes represents the mean, endpoints of vertical lines are limited to a maximum of 1.5 times the interquartile range, and ♦ represents the outliers.

NT-proBNP values of the CG and BOAS grades 0–3 pugs before and after exercise are shown in Figure 1. Three dogs had values above 900 pmol/l (two dogs in the BOAS grade 2 group and one dog in the CG). A small correlation between NT-proBNP values at rest with body weight was observed (correlation coefficient = −0.29). There was no correlation between NT-proBNP measurements with age, LA/Ao or LVIDd.

## 4. Discussion

The aim of this study was to explore BP and NT-proBNP levels in pugs with different degrees of BOAS and to compare them to levels in mesocephalic dogs. However, no differences between BP and NT-proBNP measurements of mesocephalic dogs and pugs of different grades of BOAS were identified. Therefore, in the present study, the severity of BOAS symptoms did not seem to have an impact on the measured values.

Regarding BP, some studies have observed significant differences between brachy- and mesocephalic dogs (8, 9), while others have not (6, 11). It has been shown that BP can differ among breeds (38), and use of different measurement methods can have a significant influence on the measurement results, which limits the comparability between studies including the comparison of the present study with previous studies (39, 40). The mean SBP of pugs obtained during the first measurement in the present study was comparable to mean values of the two mentioned studies in which no difference between meso- and brachycephalic dogs could have been detected (6, 11). In one of these studies, pugs were included in the brachycephalic group (11).

In one of the former studies, which detected a significant difference between BP of brachy- and mesocephalic dogs (8), mesocephalic dogs had lower mean (±SD) (153.5 ± 21.7) values than brachycephalic dogs (177.6 ± 25.0), but values of the mesocephalic dogs were still higher than the range considered normotensive (<140 mmHg) (16). These findings were also higher than values of meso- and brachycephalic dogs found in the present study. As a possible explanation for the high BP values in the brachycephalic group in this former study, a mechanism similar to OSA in humans was presumed (8).

In studies concerning OSA in humans, intermittent hypoxia led to an activation of the renin-angiotensin system, sympathetic nervous system, endothelial dysfunction and thus an increased risk for hypertension (41, 42). Since a breathing pattern similar to OSA-affected humans has been shown in English Bulldogs (15), a similar mechanism is hypothesised causing an increased risk for hypertension (8, 9). However, studies indicated that there are breed-related differences in symptoms of BOAS (2, 37, 43, 44). As results in previous studies, which found differences between brachy- and mesocephalic dogs were not divided by breed, breed-related variations among the different brachycephalic breeds cannot be ruled out. This would be a possible explanation for the different results in the studies by de Melo Dias et al. (9) and Hoareau et al. (8) and the results presented here.

In the present study, BP values in all groups were lower during the second measurement. Since this measurement was performed at the end of the examination day, it is likely that the dogs were more familiar with the environment and investigators at that time. A familiarisation also affected the behaviour and stress level of the dogs, since most dogs (84%) were relaxed during the second measurement, while the majority of dogs (66%) were slightly nervous during the first measurement. The influence of different settings and stress level on BP values has been shown in several studies (45–48). The results of the present study emphasise the need to consider the settings when interpreting BP values, whereby this factor seemed to have a greater impact on BP levels than breed or clinical signs of BOAS in the present study.

The mean values of each group were below 900 pmol/l, which is the recommended cut-off value for the differentiation between dyspnoea related to cardiac or non-cardiac disease (49). Two pugs in the BOAS grade 2 group and one dog in the CG had slightly higher values than 900 pmol/l but with a mean (range) of 1071.60 (942.0–1192.0) pmol/l just slightly above the cut-off value. In these dogs, no explanations for the higher values were found in the physical examination, echocardiography or blood test. When comparing NT-proBNP values with those from other studies in which the same test (canine Cardiopet^®^ proBNP ELISA, IDEXX Laboratories, Ludwigsburg, Germany) was used, the results were in the same range as the healthy control groups (21, 50).

In the present study, NT-proBNP values in all groups were higher after exercise. Exercise-induced increases in NT-pro BNP levels have been shown in humans (51, 52) and also in dogs after submaximal exercise (21). Despite the increase in NT-proBNP levels through exercise in this study, the mean values were below the laboratory recommended cut-off value. Since no relevant differences between the groups occurred after exercise and the increase within the individual groups was small, the measurement after exercise did not seem to provide additional information for dogs in the CG and pugs in this study.

Another cardiac biomarker that was investigated in one study in the context of brachycephaly and BOAS is cTnI (10). In the study by Planellas et al. (10), 47.8% of the brachycephalic dogs had increased cTnI levels. The authors of this previous study further hypothesised that BOAS may lead to myocardial damage, whereas no significant association between cTnI levels and severity of respiratory signs could be detected. It is noticeable that increased levels only occurred in English and French Bulldogs but in none of the examined pugs (10). Both, the abovementioned study as well as findings in the present study in pugs may indicate that cardiac biomarkers may be more susceptible to breed-specific BOAS characteristics than an overall estimation of clinical BOAS severity.

Limitations of the present study include the small sample size of the groups, especially of the CG and the BOAS grade 3 group. However, the sample size of 34 pugs exceeds sample sizes of previous studies in brachycephalic dogs with regard to BP (6, 8, 9, 11). Additionally, a sample size of 42 pugs for analysing NT-proBNP is comparable to the existing study on brachycephalic dogs and cTni levels, which included 50 brachycephalic dogs, of which 29 dogs were French Bulldogs (10). Moreover, severity of BOAS symptoms was evaluated using a functional BOAS grading based on physical examination, but there was no assessment of anatomic structures under general anaesthesia. Furthermore, since the first BP measurement took place during echocardiography and the dogs therefore could not choose their position, this could also have contributed to higher values in addition to the unfamiliar environment.

In conclusion, BP and NT-pro BNP values did not differ between mesocephalic dogs and pugs with different degrees of severity of BOAS in the present study. Moreover, results of the current study indicate that the commonly used reference ranges for BP and NT-proBNP are applicable in pugs. In the present study, different levels of severity of BOAS do not seem to affect BP and NT-proBNP values. Rather, clinical settings and stress level of the dogs seem important when interpreting BP values. A clinical implication of this regarding NT-proBNP is that the cardiac biomarker may be used to differentiate between cardiac and non-cardiac respiratory distress even in pugs with clinical symptoms of BOAS. In the present study population, no association was found between BOAS symptoms in pugs and hypertension.

## Data availability statement

The raw data supporting the conclusions of this article will be made available by the authors, without undue reservation.

## Ethics statement

The animal study was reviewed and approved by Lower Saxony State Office for Consumer Protection and Food Safety (LAVES). Written informed consent was obtained from the owners for the participation of their animals in this study.

## Author contributions

IN, J-PB, PW, and RM conceived the study and participated in its design and coordination. PW and RM performed and assisted in all examinations. J-PB performed all echocardiographic examinations. RM made substantial contributions to acquiring, analysing, and interpreting the data and wrote the original draught. IN was involved in critically revising the manuscript for important intellectual content and made substantial contributions to its conception and design. MB made substantial contributions to statistical analysis and was involved in revising the manuscript for important intellectual content. J-PB, PW, and CL made substantial contributions to conception and design, analysis, and data interpretation and were involved in critically revising the manuscript for important intellectual content. All authors have read and approved the final manuscript.
