# Supplementary material for: Evaluation of blood pressure and NT-proBNP in pugs with and without clinical signs of Brachycephalic Obstructive Airway Syndrome
Source: Front Vet Sci. 2022 Dec 22;9:1015157. doi: 10.3389/fvets.2022.1015157 (PMC9815440; doi:10.3389/fvets.2022.1015157)
Supplement: Supplementary file 1 [file Table_1.DOCX]

Supplementary Material 1

Table: Functional BOAS grading based on respiratory signs before and after a submaximal fitness test (FT)

|  |  | Respiratory noise^a^ | Inspiratory effort^b^ | Dyspnoea/Cyanosis/Syncope^c^ |
| --- | --- | --- | --- | --- |
| Grade 0 | Pre-FT | Not audible | Not present | Not present |
|  | Post-FT | Not audible | Not present | Not present |
| Grade 1 | Pre-FT | Not audible or mild | Not present | Not present |
|  | Post-FT | Mild | Not present to mild | Not present |
| Grade 2 | Pre-FT | Mild to moderate | Mild to moderate | Not present |
|  | Post-FT | Moderate to severe | Moderate to severe | Mild Dyspnoea; cyanosis or syncope not present |
| Grade 3 | Pre-FT | Moderate to severe | Moderate to severe | Moderate to severe Dyspnoea; may or may not present cyanosis; inability to exercise |
|  | Post-FT | Severe | Severe | Severe Dyspnoea; may or may not present cyanosis or syncope |

The clinical grading was based on respiratory signs before (pre-FT) and after a submaximal fitness test (post-FT) corresponding to Liu et al. 2015 (1) and modified to match the present study design. During the FT, dogs trotted for fifteen minutes in an individual comfort speed (between four to eight kilometres per hour).

^a^Respiratory noise was categorised by auscultation. Mild: only audible under auscultation; moderate: intermittent audible noise that can be heard without stethoscope; severe: constant audible noise that can be heard without stethoscope.

^b^Inspiratory effort was categorised by evidence of increased inspiratory effort (inhale air with the use of diaphragm and/or accessary muscles of respiration and/or nasal flaring). Mild: regular breathing patterns with minimal use of diaphragm; moderate: evidence of use of diaphragm and accessary muscles of respiration; severe: marked movement of diaphragm and accessary muscles of respiration.

^c^Dogs that have had episodes of syncope and/or cyanosis as documented by owner's report are classified into Grade 3 without FT. Mild Dyspnoea: presents sign of discomfort; moderate Dyspnoea: irregular breathing, signs of discomfort; severe Dyspnoea: irregular breathing with sings of breathing discomfort and difficulty in breathing.

1. Liu NC, Sargan DR, Adams VJ, Ladlow JF. Characterisation of Brachycephalic Obstructive Airway Syndrome in French Bulldogs Using Whole-Body Barometric Plethysmography. PloS one. 2015;10(6):e0130741.
