# Supplementary figures and images for: Evaluation of blood pressure and NT-proBNP in pugs with and without clinical signs of Brachycephalic Obstructive Airway Syndrome
Source: Front Vet Sci. 2022 Dec 22;9:1015157. doi: 10.3389/fvets.2022.1015157 (PMC9815440; doi:10.3389/fvets.2022.1015157)

Supplementary Material Figure 1


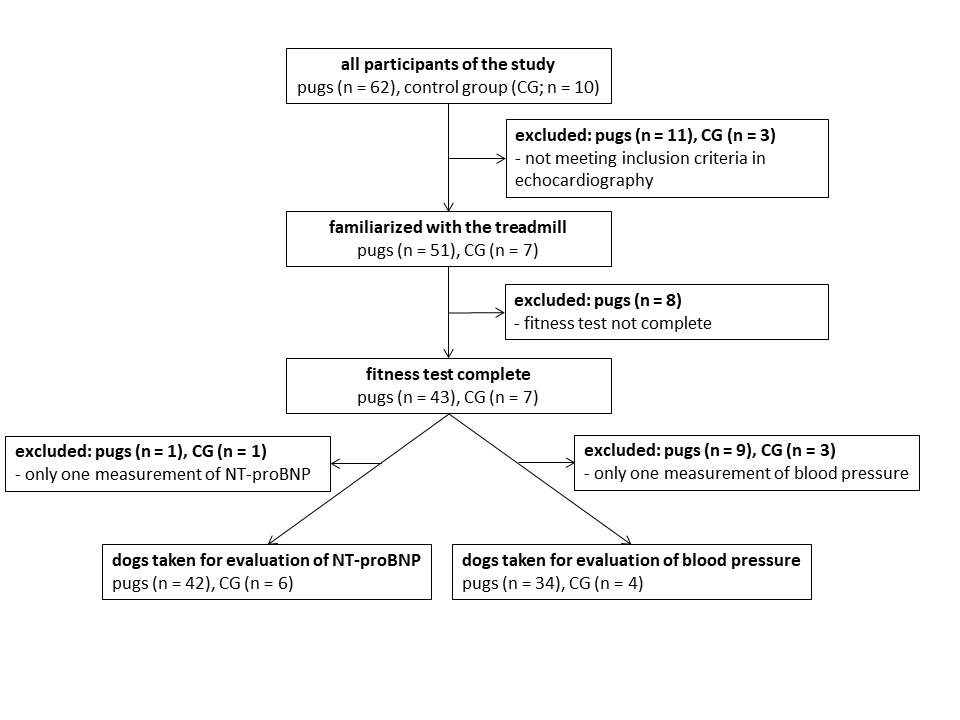


Figure 1: Overview of exclusion.

Supplement: Supplementary file 3 [file Data_Sheet_1.zip › Supplementary Figure 1.docx]
